# A single fungal strain was the unexpected cause of a mass aspergillosis outbreak in the world’s largest and only flightless parrot

**DOI:** 10.1016/j.isci.2022.105470

**Published:** 2022-11-02

**Authors:** David J. Winter, Bevan S. Weir, Travis Glare, Johanna Rhodes, John Perrott, Matthew C. Fisher, Jason E. Stajich, Kākāpō Aspergillosis Research Consortium, Andrew Digby, Peter K. Dearden, Murray P. Cox

**Affiliations:** 1 School of Natural Sciences, Massey University, Palmerston North, New Zealand; 2 Genomics Aotearoa, University of Otago, Dunedin, New Zealand; 3 Manaaki Whenua – Landcare Research, Auckland, New Zealand; 4 Department of Wine, Food & Molecular Bioscience, Lincoln University, Lincoln, New Zealand; 5 MRC Centre for Global Infectious Disease Analysis, Imperial College School of Public Health, Imperial College London, South Kensington, London SW7 2BX,UK; 6 School of Environmental Science, Faculty of Health and Environmental Science, Auckland University of Technology, Auckland, New Zealand; 7 Department of Microbiology and Plant Pathology, University of California, Riverside, Riverside, Riverside, CA, USA; 8 Department of Biochemistry, University of Otago, Dunedin, New Zealand; 9 Kākāpō Recovery, Department of Conservation, Invercargill, New Zealand

**Keywords:** Animals, Microbiology, Microbiology parasite, Parasitology

## Abstract

Kākāpō are a critically endangered species of parrots restricted to a few islands off the coast of New Zealand. Kākāpō are very closely monitored, especially during nesting seasons. In 2019, during a highly successful nesting season, an outbreak of aspergillosis affected 21 individuals and led to the deaths of 9, leaving a population of only 211 kākāpō. In monitoring this outbreak, cultures of aspergillus were grown, and genome sequenced. These sequences demonstrate that, very unusually for an aspergillus outbreak, a single strain of aspergillus caused the outbreak. This strain was found on two islands, but only one had an outbreak of aspergillosis; indicating that the strain was necessary, but not sufficient, to cause disease. Our analysis provides an understanding of the 2019 outbreak and provides potential ways to manage such events in the future.

## Highlights

• In 2019, the kākāpō, an endangered parrot species, was threatened by aspergillosis
• The outbreak was associated with a single strain of *Aspergillus fumigatus*
• The first reported case of a single strain of *Aspergillus* causing a disease outbreak

## Graphical abstract

**Figure:**
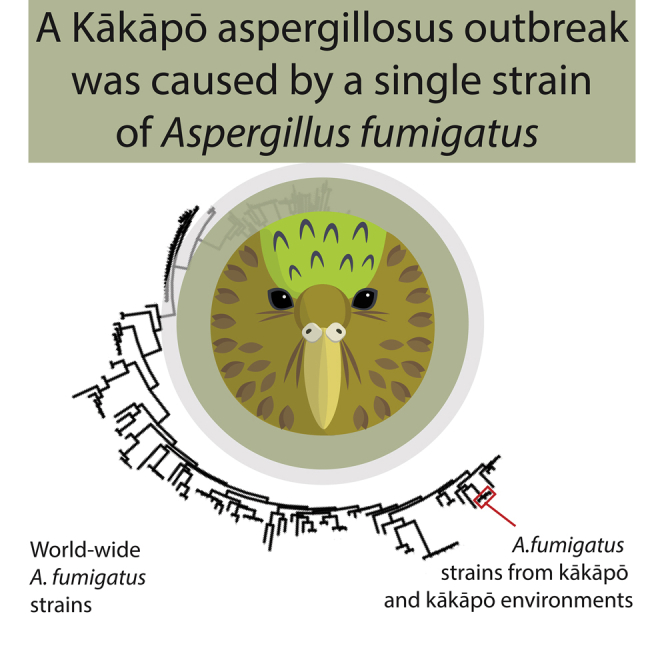

## Introduction

Species of conservation concern, even when carefully managed, are always vulnerable to new infectious diseases. If not managed and understood, these diseases can rapidly cause species with critically small populations to become inviable or even go extinct. The kākāpō (*Strigops habroptilus*), the world’s largest parrot, is nocturnal, flightless, and endemic to New Zealand. Once widespread, kākāpō were almost extirpated by mammalian predators after human settlement of the islands.^1^^,^^2^^,^^3^^,^^4^ The species is now critically endangered, comprising around 200 individuals restricted to five offshore sanctuary islands.^5^ The survival of these remaining populations of kākāpō depends on an intensive conservation management program, which aims to mitigate threats such as high infertility and disease^6^ and maintain the islands in a predator-free state. Diseases affecting kākāpō include erysipelas,^7^ vent dermatitis,^8^ exudative cloacitis,^9^ aflatoxicosis (caused by Aspergillus fungi),^10^ and the focus of this study, aspergillosis.

During the unprecedentedly successful breeding season of kākāpō in 2019, an outbreak of aspergillosis affected 21 individuals (∼10% of the total species census), killing nine nesting females, chicks and juveniles, and leaving only 211 individuals living at the time. This outbreak was limited to just one of the offshore islands, Whenua Hou (also known as Codfish island), with no cases occurring elsewhere. All but two of the cases (both discovered postmortem) occurred during a very short window of time between April and May 2019. The effects of this outbreak would have been even greater if not for a remarkable and intensive effort to diagnose and treat kākāpō at risk, involving transfers of 51 kākāpō to veterinary facilities on the mainland and computed tomography (CT) scans of 45 of these birds^11^ (see Table S1). Although this rescue from aspergillosis was successful, it is of utmost importance to minimize the risk of such outbreaks in the future, in the kākāpō and other threatened birds. It, therefore, became a priority to determine the source of the infection, explore the reasons for the outbreak, and identify factors that might mitigate its recurrence in the future.

Aspergillosis is a respiratory disease caused by the inhalation of spores from fungi in the genus *Aspergillus*.^12^ The disease is most commonly caused by *A. fumigatus*, a genetically diverse species that is globally near ubiquitous in soil and decaying plant matter.^13^ Despite frequent exposure to *Aspergillus* spores, the development of aspergillosis in mammals, including humans, is typically limited to immunocompromised and immunosuppressed individuals. However, the lung physiology of birds makes them much more susceptible, and aspergillosis can affect otherwise healthy individuals when spore loads are high.^14^ Outbreaks of aspergillosis have been recorded in free-ranging birds including gulls, raptors and waterfowl,^15^^,^^16^ as well as in managed populations of other New Zealand endemic birds, such as hihi (*Notiomystis cincta*, stitchbird)^17^ and kiwi.^18^ These outbreaks are typically associated with environmental changes that encourage the growth of *Aspergillus* and expose individuals to very high concentrations of spores.^19^ There had previously been only one recorded case of aspergillosis in kākāpō, leading to the death of a juvenile, named Rooster, on Whenua Hou in 2012 (see Table S2). The 2019 outbreak was therefore a unique, and unexpected, event.

Genome-wide epidemiological approaches allow researchers to study the origin and dynamics of disease outbreaks with great detail.^20^^,^^21^ Whole-genome sequencing of organisms responsible for a disease outbreak can reveal the evolutionary and molecular basis of the disease, helping to identify the source of an outbreak and to inform efforts to limit or eradicate the problem in the future. The 2019 aspergillosis outbreak in kākāpō jeopardized an entire species of international conservation importance. It threatened the survival of a large proportion of the population, and severely disrupted reproduction at one of the most important breeding sites for the species. Similar events in the future could disrupt the intensive management on which kākāpō population growth is dependent, so there is an urgent need to determine the characteristics and source of this outbreak. With aspergillosis common in birds, this knowledge is also of significance to a wide range of threatened avian species which are dependent on both *in situ* and *ex situ* conservation. We have therefore characterized the pathogen responsible for the 2019 kākāpō aspergillosis outbreak and identified widely applicable learnings for best conservation practice.

## Results

### Extensive sampling of infected individuals and environmental sites

**Figure 1:**
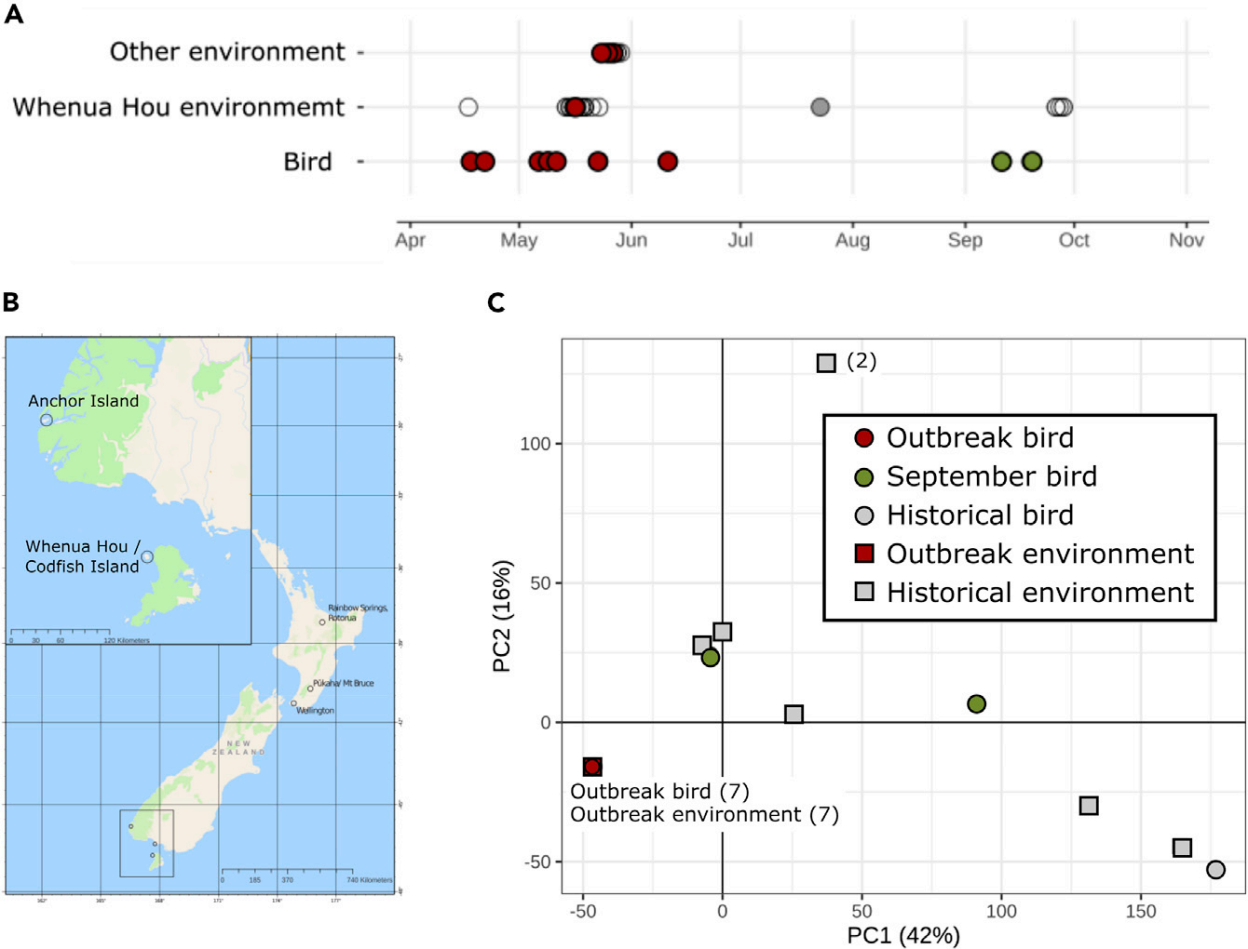
A single low-diversity *A. fumigatus* lineage was responsible for the aspergillosis outbreak (A) Timeline of sampling relating to the 2019 outbreak. Each point represents a sample, filled circles represent samples from which *A. fumigatus* could be cultured. (B) Map indicating the locations of *A. fumigatus* isolates in New Zealand. (C) Principal component analysis of SNP data including all New Zealand isolates. Environmental isolates are represented by squares, bird-derived isolates by circles. All isolates taken from birds during the outbreak occupy the same position in this figure so are represented by a single point.

We obtained fungal cultures from all nine kākāpō that succumbed to the outbreak (Figure 1 and Tables S1 and S2). To compare these isolates with natural environmental diversity on Whenua Hou and more widely across New Zealand (Figure 1B), we also attempted to culture *Aspergillus* from 68 environmental sites. Given the restricted geographical distribution of the outbreak, our sampling focused on Whenua Hou (46 samples) and Anchor Island (another kākāpō sanctuary 150 km away that did not experience an aspergillosis outbreak; 22 samples).

Most of the samples were collected during the peak of the outbreak, with follow-up sampling occurring later in the year (Figure 1A). This sampling strategy allowed us to identify the strains responsible for the disease outbreak, and to investigate potential sources of these infections by comparison to environmental isolates taken from Whenua Hou. We were also able to compare strains present on Whenua Hou during the outbreak to outbreak-free Anchor Island and the diversity of *Aspergillus* observed across New Zealand more widely.

The majority of the environmental samples did not contain culturable *Aspergillus*. In total, we obtained *Aspergillus* isolates from seven environmental samples, four from Whenua Hou and three from Anchor Island (Figures 1A and Table S2). Finally, to compare isolates collected during the outbreak with others present in New Zealand more generally, we obtained seven historical *A. fumigatus s*amples from the ICMP culture collection (International Collection of Microorganisms from Plants; https://www.landcareresearch.co.nz/icmp).

### The peak of the outbreak was caused by a single *A. fumigatus* strain

Sequencing the internal transcribed spacer (ITS) locus in *Aspergillus* isolates cultured from kākāpō revealed that all *Aspergillus* infected birds shared a single haplotype, which also matches many *A. fumigatus* isolates in the ICMP collection (Table S2). This identified the species responsible for the outbreak, but whole-genome resequencing was needed to uncover genetic patterns among these isolates at a finer scale. Our sequencing approach yielded high coverage (>39x) short-read sequence data for 24 strains from across New Zealand. With an average of 95% of sites called per sample, we identified 169,997 polymorphic single nucleotide variants (SNVs) in this dataset.

**Figure 2:**
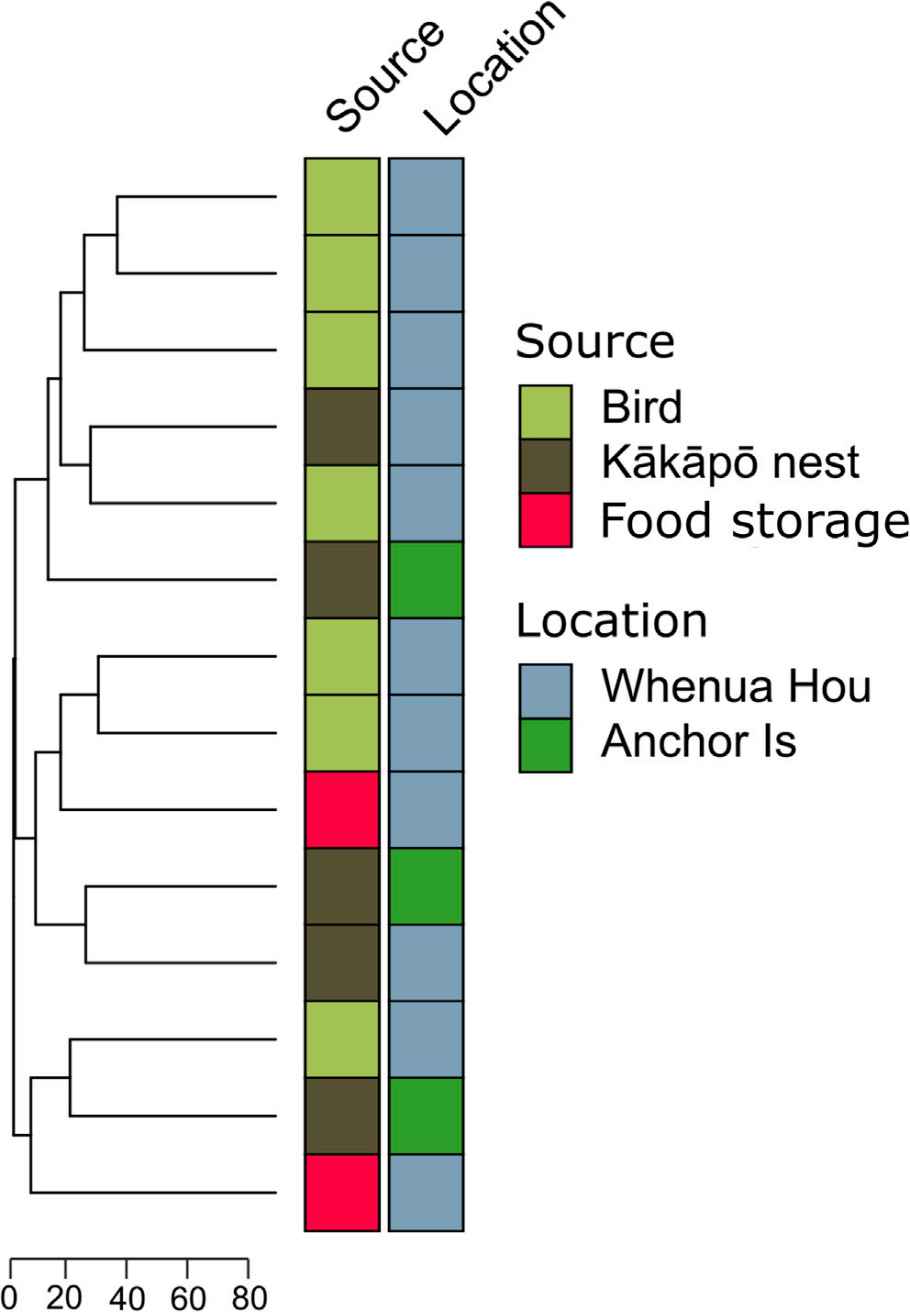
Genetic data cannot differentiate environmental from bird-derived isolates Hierarchical clustering was used to compare isolates from the outbreak strain. The scale bar represents the total number of SNP differences between given stains. The shaded boxes represent metadata associated with each isolate, specifically the source of each isolate and the island from which each isolate was recovered.

A principal component analysis (PCA) performed on SNV genotypes (Figure 1C) demonstrates that isolates from kākāpō infected during the peak of the outbreak were part of a very closely related group. This single strain, represented by the red dot on the PCA plot, occurs in all 7 birds that died during the peak of the outbreak and was also found in environmental sites on both Whenua Hou (containing 52 adults, 14 juveniles and 33 chicks as of 1/4/2019), where the outbreak occurred and Anchor Island (containing 38 adults, 21 juveniles and 28 chicks as of 1/4/2019), where it did not (Figure 2). These genomes, both the environmental samples and those collected from kākāpō that died between April and June 2019, differed from each other at only 3.5–4 sites per million bases pairs, which is within the margin of SNV detection error. The genomes in the outbreak strain are therefore essentially identical.

The low diversity of the strain infecting birds early in the outbreak is a striking result. To put the diversity of the outbreak strain isolates in context, we used our pipeline to call SNVs from two human clinical isolates taken from the same patient on the same day. Those samples differed at 6.1 sites per million base pairs. Thus, the outbreak strain isolates taken from birds and environmental samples on Whenua Hou and Anchor Island in 2019 were more similar to each other than samples taken from a single infected patient. The dominance of a single low-diversity strain in these locations is a classic sign of a clonal expansion, the rapid spread of a fast-growing strain that supplants existing lineages or fills a vacant niche. Although very common with other infectious diseases, avian aspergillosis is invariably caused by high fungal loads of multiple *Aspergillus* strains. A sudden outbreak of aspergillosis caused by a single *A. fumigatus* strain has not previously been recorded.

The main aspergillosis outbreak peaked in May 2019, but two juvenile birds died of aspergillosis in September later in the same year. *A. fumigatus* was cultured from these two birds and sequenced. The isolates recovered were genetically distinct from those infecting birds during the peak of the outbreak, with one closely related to strains collected from kiwi nests in mainland New Zealand and the other having no close relatives in our dataset (Figure 1C). The primary outbreak event, therefore, appears to have been tightly restricted, both temporally to April-May 2019 and geographically to Whenua Hou.

### The outbreak strain was widely distributed at kākāpō sites

Hierarchical clustering of SNV genotypes from the outbreak strain did not distinguish between isolates recovered from infected birds and environmental samples, or between isolates taken from different islands (Figure 2). The strain was found in environmental samples from diverse sites associated with kākāpō management on both Whenua Hou and Anchor Island, including nests, track and bowl display sites,^22^ feeding stations, and buildings used for the preparation and storage of supplementary food. No other *A. fumigatus* strains were identified on the island between April and July.

Importantly, isolates from Anchor Island were not genetically distinct from those on Whenua Hou, even though no disease was recorded on Anchor Island. This suggests the isolates infecting birds do not represent a distinct lineage of the outbreak strain present on Whenua Hou Island. Similarly, management and feeding practices were the same on both islands. The mere presence of this strain on an island does not in itself ensure that an outbreak will occur.

#### The outbreak strain is not closely related to any previously characterized strain

**Figure 3:**
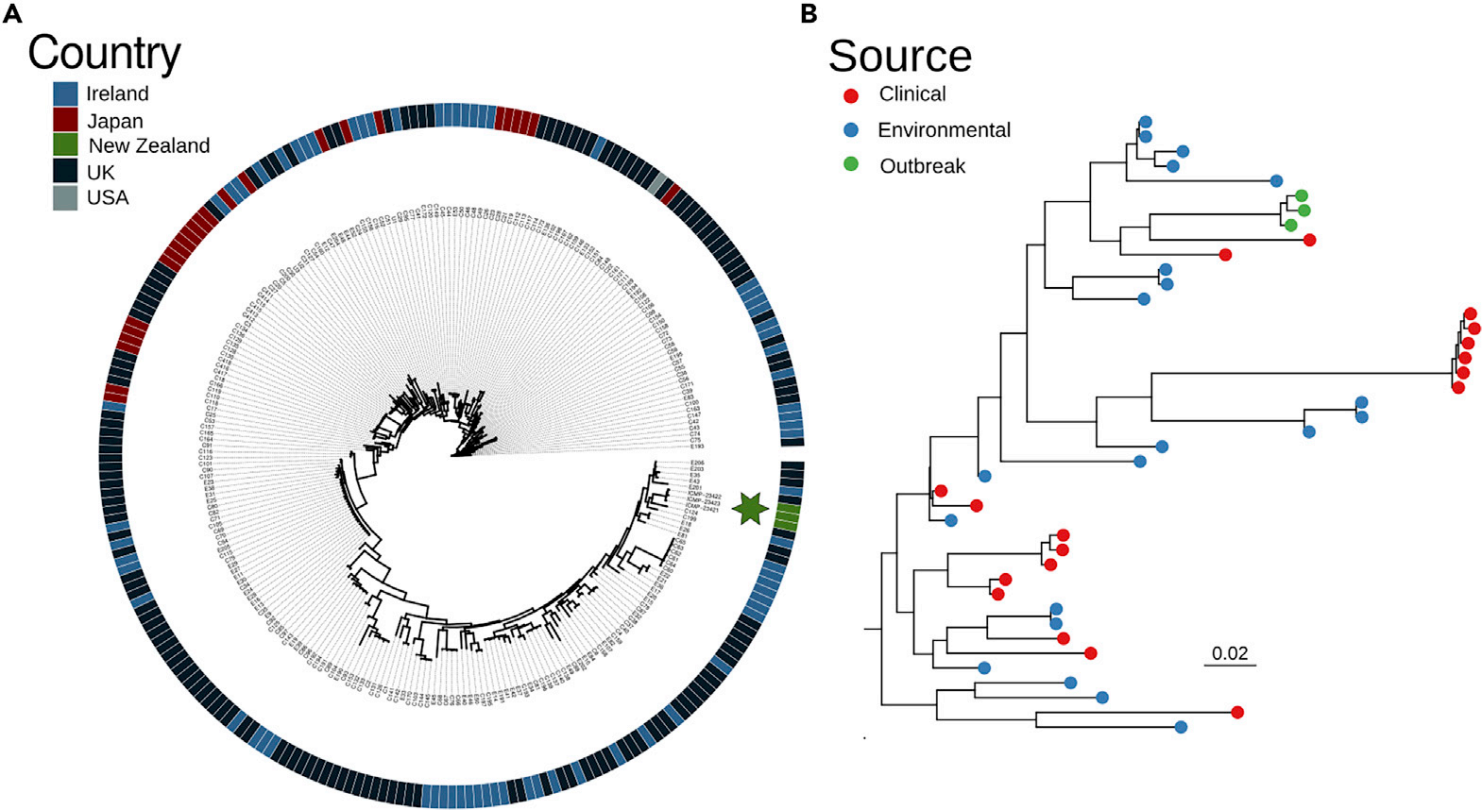
The outbreak strain is not closely related to other sequenced strains (A) The outbreak strain is included in a global phylogeny of *A. fumigatus*. Outbreak strains are shaded green and noted by a star. Isolates from other countries are shaded following the figure legend. (B) A subtree extracted from A, with tips shaded to reflect the source of each isolate (with human clinical isolates in red and environmental isolates in blue). The outbreak strain is most closely related to human clinical strains, but part of a wide clade that includes many environmental isolates.

We next considered whether the origin of the outbreak strain could be determined by comparing its sequence to the sequences of other *A. fumigatus* strains. We built a phylogeny using our sequences and a database of the global diversity of *A. fumigatu*s (Figure 3).^23^

The outbreak strain forms a clade with two clinical isolates collected from the UK and Ireland. This clade is in turn nested within a wider grouping containing mostly environmental isolates. The database used for this comparison is strongly biased toward samples from the UK and Ireland (218 of 247 samples in our subtree),^23^ meaning the geographic origin of the outbreak strain cannot be determined using the currently available genomic data, and further sampling is required. However, the close relationship between the outbreak strain and two clinical isolates may suggest that the outbreak strain is pre-adapted to growth in a vertebrate host.

##### Virulence genes in the outbreak strain are largely inconclusive

*A. fumigatus* is an opportunistic pathogen, and therefore not primarily adapted to growth within an animal host. Nevertheless, strains differ in their ability to establish infections and cause disease,^24^^,^^25^ suggesting that some genotypes are more virulent than others. Although no easily definable ‘virulence factors’ are known for *A. fumigatus*, certain biological functions and classes of genes have been demonstrated to contribute to pathogenesis.^26^ We find 138 of 240 putative virulence genes contain non-synonymous mutations when the outbreak strain is compared to the Af293 reference genome. In addition, the most significant protein-coding change from the reference genome is *cyp5081A1,* a gene in a secondary metabolite-producing cluster, which has acquired an in-frame stop leading to a severe truncation of the protein product. *Cyp5081A1* encodes a cytochrome p450 enzyme thought to play a role in helvolic acid biosynthesis,^27^ implying this strain may be deficient in this antibacterial product. We also produced a *de novo* assembly for sample ICMP 23423 to check for gene duplications, which would be difficult to detect from our genotyping data but found no evidence for duplications in putative virulence genes.

The outbreak strain has a wild type allele for *cyp51A*, a gene whose derived variants are associated with resistance to azole treatment of *A. fumigatus*. Consistent with this, azole treatment proved highly effective as a clinical therapeutic during this outbreak. Although we can determine the genotypes of putative virulence-associated genes, it is not currently possible to predict the phenotypic effects of these alleles, especially given the very large number of them. The functional basis of the outbreak, assuming it even has a genetic cause, therefore remains unclear.

## Discussion

The 2019 outbreak of aspergillosis in kākāpō had a significant impact on this critically endangered species. Nine birds (two adults, five chicks and two juveniles) died, a substantial loss for a species comprising only 115 adults at the time. The impact on nesting females was of particular concern, given the slow reproductive rate of this species. The outbreak may have been much worse if not for an intensive and extensive mitigation effort, which included widespread blood testing and translocation of over 50 individuals to veterinary hospitals for diagnostics and treatment. Before this outbreak, only a single case of aspergillosis was known in kākāpō, suggesting that special conditions may have contributed to the 2019 event. Given the potential future impact on kākāpō and other threatened bird species, and the huge expense required to manage the disease, it is crucial that we better understand the nature of this outbreak.

We determined that all infections during the peak of the 2019 outbreak were caused by a single strain of *A. fumigatus*. Whole-genome population genetic data show that the strain causing the outbreak has exceptionally low genetic diversity and is distinct from *A. fumigatus* isolates present on mainland New Zealand. This is counter to findings that avian epizootic events are typically driven by diverse strains.^28^ Importantly, the only historical isolate from Whenua Hou (collected from a kakapo in 2012) is more closely related to mainland New Zealand isolates than the outbreak strain. This result suggests the outbreak strain is not simply the only *A. fumigatus* lineage historically present on Whenua Hou.

Because a single strain was found in all the kākāpō in the initial outbreak, and that avian aspergillosis outbreaks are usually caused by diverse strains, it is tempting to speculate that this strain has a particularly infective genotype. However, we find the same strain was present in kākāpō nests and a track-and-bowl mating site, on nearby Anchor Island, where no aspergillosis outbreak occurred. The presence of this strain is not by itself enough to cause the disease. Moreover, two birds that succumbed to the disease following the peak of the outbreak were infected by very different *A. fumigatus* lineages. These results combine to show that the ‘outbreak strain’ was neither sufficient nor necessary for the development of aspergillosis. Nevertheless, we do note that the strain at the root of the outbreak contains several non-reference alleles in genes that may contribute to pathogenesis in *A. fumigatus*, and there is ongoing work to phenotype this strain and test whether it may be more virulent than other strains.

The presence of extremely closely related strains on Whenua Hou and Anchor Island suggests a common origin. Aspergillosis outbreaks in birds have been linked with environmental conditions that favor the growth of *Aspergillus*.^19^ The summer of 2019 saw a combination of an unusually warm and dry summer (https://niwa.co.nz/climate/our-services/virtual-climate-stations), and the production of rimu fruit (*Dacrydium cupressinum*; a primary breeding food source for kākāpō) on both Whenua Hou and Anchor Island. In addition, there was only the second instance in 35 years of records of rimu fruit ripening on Whenua Hou. We suggest that the 2019 aspergillosis outbreak may have been a ‘black swan’ event, in which multiple variables combined to cause the disease. These may include a more virulent strain of *A. fumigatus* in the island environment, and a warm, dry summer favoring fungal spore production in soil and leaf litter, as well as causing dusty conditions that increase airborne spore densities.^15^ Given environmental sampling for Aspergillus fungus is not normal routinely carried out, it is unclear if the detections reported here are unusual, reflecting the unusual summer, or within a normal range.

One important possibility is that the strain was human-spread. Kākāpō are intensively managed and supplemental feeding, of commercially sourced food, is carried out during the breeding period. A link between supplementary food consumption and aspergillosis infection provides a possible source of the strain (Kākāpō Recovery Program, unpublished data), although the absence of an outbreak on equally managed Anchor Island does not make a human-mediated cause entirely straightforward. The finding of the outbreak strain in food storage areas implies that if this was not the origin of the strain, it was contaminated, and may have been involved in the spread of the strain.

That extremely closely related strains were present on both kākāpō breeding islands, but disease only occurred on Whenua Hou, is instructive in determining the likely disease etiology. Aspergillosis usually occurs when fungal loads are very high, or immuno-compromised individuals are exposed to substantial amounts of *Aspergillus*. These conditions were therefore apparently met on Whenua Hou but not Anchor. This is in accordance with the occurrence of another kākāpō disease, cloacitis,^8^ on Whenua Hou but not on Anchor.

The potential that the strain was introduced to the kākāpō islands by human activity is of extreme concern for conservation management. Both islands are subject to strict quarantine and hygiene procedures, including the application of an anti-fungal disinfectant to all equipment and clothing transported to the islands, and regular disinfection of supplementary feeding equipment. This reduces the likelihood of the transfer of fungal spores between islands but does not eliminate the risk. Once on the islands, the movement of chicks and equipment or the provision of supplementary food may have provided a mechanism for the transfer of the strain between nests, even though this equipment is subject to disinfection protocols.

Our results indicate that the virulent strain may have been introduced to the kākāpō islands through human activity, but its effect was exacerbated by exceptional weather conditions that summer. Using whole-genome sequencing data to understand this epidemic provides key data and allows us to suggest procedures in place to detect and mitigate such events in the future. Identification of *Aspergillus* strains via genetic techniques, such as PCR or Oxford Nanopore sequencing, could rapidly indicate when problems might occur and be used to guide adaptive management. For example, detection of potentially pathogenic *Aspergillus* isolates could trigger measures such as enhanced hygiene controls, limits on the movement of eggs, chicks and equipment among nests and islands, and widespread screening for Aspergillosis. We suggest the routine PCR screening of supplemental food, or other materials that come in direct contact with kākāpō, to ensure that contamination is readily and rapidly detected during breeding seasons. Positive tests could be followed up with growth assays, and then possibly genome sequencing. This ability to rapidly detect potentially pathogenic strains is of importance to a range of species subject to conservation management that are susceptible to Aspergillosis, such as hihi and kiwi in New Zealand. Furthermore, the warm dry summer conditions which may have contributed to this outbreak will become more common with global warming, making genetic surveillance for disease-causing strains a crucial tool for managing the conservation of threatened species.

### Limitations of the study

This study, led by the genomics team within the Kākāpō Aspergillosis Research Consortium, has naturally focused on genetic analysis of the *A. fumigatus* strains found in infected birds and associated environmental samples. Although most details about the outbreak have already been documented in Buchanan (2020),^11^ research by the veterinary team includes extensive CT and other diagnostic data, which are the subject of a series of forthcoming papers. Similarly, the microbiology team has screened for novel viruses, explored the broader Kākāpō microbiome, and characterized physiological activity of the *Aspergillus* strains reported here. That work is also being prepared for upcoming publication. The researchers in these related teams are authors on this study under the Kākāpō Aspergillosis Research Consortium banner, and on the basis of extensive communication between all the teams, those related analyses are consistent with the information presented here. Nonetheless, analysis of the 2019 outbreak is ongoing, and it remains possible that it was exacerbated by currently unknown factors; for example, an unidentified viral infection that led to reduced immunity in the birds, thus allowing for their infection by a widespread strain of *A. fumigatus*. So far, no cause beyond the single *Aspergillus* strain identified here has been identified, but we remain open to other possibilities.

## STAR★Methods

### Key resources table

**Table:** 

REAGENT or RESOURCE	SOURCE	IDENTIFIER
**Biological samples**		

*Aspergillus fumigatus* fungal strain	https://scd.landcareresearch.co.nz/	ICMP23421
*Aspergillus fumigatus* fungal strain	https://scd.landcareresearch.co.nz/	ICMP23422
*Aspergillus fumigatus* fungal strain	https://scd.landcareresearch.co.nz/	ICMP23423
*Aspergillus fumigatus* fungal strain	https://scd.landcareresearch.co.nz/	ICMP23424
*Aspergillus fumigatus* fungal strain	https://scd.landcareresearch.co.nz/	ICMP23425
*Aspergillus fumigatus*fungal strain	https://scd.landcareresearch.co.nz/	ICMP23437
*Aspergillus fumigatus* fungal strain	https://scd.landcareresearch.co.nz/	ICMP23479
*Aspergillus fumigatus* fungal strain	https://scd.landcareresearch.co.nz/	ICMP23465
*Aspergillus fumigatus* fungal strain	https://scd.landcareresearch.co.nz/	ICMP23467
*Aspergillus fumigatus* fungal strain	https://scd.landcareresearch.co.nz/	ICMP23468
*Aspergillus fumigatus* fungal strain	https://scd.landcareresearch.co.nz/	ICMP23469
*Aspergillus fumigatus* fungal strain	https://scd.landcareresearch.co.nz/	ICMP23470
*Aspergillus fumigatus* fungal strain	https://scd.landcareresearch.co.nz/	ICMP23472
*Aspergillus fumigatus* fungal strain	https://scd.landcareresearch.co.nz/	ICMP23474
*Aspergillus fumigatus* fungal strain	https://scd.landcareresearch.co.nz/	ICMP23475
*Aspergillus fumigatus* fungal strain	https://scd.landcareresearch.co.nz/	ICMP23477
*Aspergillus fumigatus* fungal strain	https://scd.landcareresearch.co.nz/	ICMP23478
*Aspergillus fumigatus* fungal strain	https://scd.landcareresearch.co.nz/	ICMP23499
*Aspergillus fumigatus* fungal strain	https://scd.landcareresearch.co.nz/	ICMP23502
*Aspergillus fumigatus* fungal strain	https://scd.landcareresearch.co.nz/	ICMP23512
*Aspergillus fumigatus* fungal strain	https://scd.landcareresearch.co.nz/	ICMP434
*Aspergillus fumigatus* fungal strain	https://scd.landcareresearch.co.nz/	ICMP1061
*Aspergillus fumigatus* fungal strain	https://scd.landcareresearch.co.nz/	ICMP23529
*Aspergillus fumigatus* fungal strain	https://scd.landcareresearch.co.nz/	ICMP23536
Metadata on *Aspergillus fumigatus* fungal strains	https://www.gbif.org/	https://doi.org/10.15468/dL.p5q66r

**Chemicals, peptides, and recombinant proteins**		

MagAttract HMW DNA Kit	Qiagen	Cat#67563
Agencourt AMPure XP	Beckman & Coulter,	Cat#A63881

**Deposited data**		

*A. fumigatus* Af293 reference	NCBI	GCA_000002655.1
*A. fumigatus* global genome dataset	Microreact	https://microreact.org/project/6KR8996ywtVRV5wm233YhP
*A. fumigatus* ICMP23421	SRA	SRR14364154
*A. fumigatus* ICMP23422	SRA	SRR14364149
*A. fumigatus* ICMP23423	SRA	SRR14364148
*A. fumigatus* ICMP23424	SRA	SRR14364147
*A. fumigatus* ICMP23425	SRA	SRR14364146
*A. fumigatus* ICMP23437	SRA	SRR14364145
*A. fumigatus* ICMP23479	SRA	SRR14364156
*A. fumigatus* ICMP23465	SRA	SRR14364144
*A. fumigatus* ICMP23467	SRA	SRR14364143
*A. fumigatus* ICMP23468	SRA	SRR14364164
*A. fumigatus* ICMP23469	SRA	SRR14364163
*A. fumigatus* ICMP23470	SRA	SRR14364162
*A. fumigatus* ICMP23472	SRA	SRR14364161
*A. fumigatus*ICMP23474	SRA	SRR14364160
*A. fumigatus* ICMP23475	SRA	SRR14364159
*A. fumigatus* ICMP23477	SRA	SRR14364158
*A. fumigatus* ICMP23478	SRA	SRR14364157
*A. fumigatus* ICMP23499	SRA	SRR14364155
*A. fumigatus* ICMP23402	SRA	SRR14364153
*A. fumigatus* ICMP23412	SRA	SRR14364152
*A. fumigatus* ICMP434	SRA	SRR14364166
*A. fumigatus* ICMP1061	SRA	SRR14364165
*A. fumigatus* ICMP23529	SRA	SRR14364151
*A. fumigatus* ICMP23536	SRA	SRR14364150

**Software and algorithms**		

bwa mem v0.7.15	Li^33^	arXiv:1303.3997v2
GATK HaplotypeCaller v3.6	McKenna et al.^34^	https://doi.org/10.1101/gr.107524.110
Ensembl Fungi variant effect predictor	McLaren et al.^36^	https://doi.org/10.1186/s13059-016-0974-4
vcftools v1.1.16	Danecek et al.^37^	https://doi.org/10.1093/bioinformatics/btr330
vcfR v1.11.0	Knaus et al.^39^	https://doi.org/10.1111/1755-0998.12549
adegenet v2.1.2	Jombart^40^	https://doi.org/10.1093/bioinformatics/btn129
poppr v2.8.6	Kamvar et al.^41^	https://doi.org/10.7717/peerj.281
SPAdes v3.11.1	Bankevich et al.^42^	https://doi.org/10.1089/cmb.2012.0021
QUAST v4.043	Bankevich et al.^42^	https://doi.org/10.1089/cmb.2012.0021
exonerate v2.2.044	Gurevich et al.^43^	https://doi.org/10.1093/bioinformatics/btt086
RAxML v8.2.9	Slater and Birney^44^	https://doi.org/10.1186/1471-2105-6-31
FigTree v1.4.2	http://tree.bio.ed.ac.uk/software/figtree/	https://github.com/rambaut/figtree/releases
Scripts to rerun analyses	https://github.com/dwinter/kakapo_aspergillosis	https://doi.org/10.5281/zenodo.7213784

### Resource availability

#### Lead contact

• Further information and requests for resources and reagents should be directed to and will be fulfilled by the lead contact, Peter K Dearden (Peter.dearden@otago.ac.nz)

#### Materials availability

• The *Aspergillus* strains used in this study are available on request from https://scd.landcareresearch.co.nz/ Accession numbers are listed in the key resources table.
• Full metadata on *Aspergillus* cultures is available at https://scd.landcareresearch.co.nz/ and on GBIF at https://doi.org/10.15468/dl.p5q66r.

### Experimental model and subject details

#### Kākāpō management information

Kākāpō (*Strigops habroptila*) are large, flightless ground-living parrots. All Kākāpō currently exist on five predator-free islands in New Zealand and are subject to intensive conservation management by the Kākāpō Recovery Team, Department of Conservation/Te Papa Atawhai. Breeding primarily occurs on two of these: Whenua Hou and Anchor Island. Every kākāpō individual wears a ‘smart’ transmitter, which is used to determine their location and monitor activity. Mating and nesting behaviour is recorded by the transmitters and relayed to conservation staff by a data network connected to the internet.

Kākāpō breed infrequently, typically only every 2-3 years, synchronized with the mass fruiting of certain tree species. On Whenua Hou and Anchor Island, rimu (*Dacrydium cupressinum*) triggers breeding attempts. Nesting typically starts in February, with nests usually in hollow logs or cavities among tree roots. If necessary, nests are modified to provide safety and weather protection or moved into adjacent nest boxes if the cavity is too small or otherwise unsuitable. Proximity sensors connected to the data network are used to remotely monitor female activity at the nest.

Kākāpō lay 1-5 eggs, which are often removed for artificial incubation and replaced by ‘dummy’ eggs. Chicks that hatch in captivity are usually returned to the nest at 1-2 days old. They are weighed and given a health check every 1-5 days by conservation staff. To optimize chick health and maximize the number of chicks in nests, cross-fostering of chicks between mothers is common, so chicks are often raised by a foster mother.

The default is that chicks are raised in nests, but some chicks are hand-reared due to illness or insufficient nest capacity. These birds are raised either in facilities on the kākāpō islands or at facilities on mainland New Zealand. The chicks are returned to weaning pens on the islands and then released into the wild at about 120 days old.

During breeding seasons, kākāpō are provided with supplementary food to optimize their breeding condition. A commercial parrot food (High Protein Coarse, Harrison’s Bird Foods, Brentwood, TN, USA) is provided in a separate feeding station for each individual. Mothers preferentially feed ripe rimu fruit to their chicks, but if that is not available (the fruit often fails to ripen), they will feed their chicks with supplementary food.

Diagnosis of Kākāpō with aspergillosis was carried out via standard clinical procedures^29^^,^^30^ as described in Buchanan (2020).^11^ Treatment of non-fatal cases is described in Buchanan (2020).^11^

#### Fungal isolates

To determine the distribution of fungal isolates, environmental samples were collected in May–June 2019 from sites associated with kākāpō management: on Whenua Hou; on Anchor Island, where breeding occurred but no aspergillosis was detected; and at the kākāpō hand-rearing facility in Invercargill. Samples of leaf litter, soil and detritus were collected from nests, near tracks and from buildings used for kākāpō supplementary food preparation and storage on Whenua Hou and Anchor Island.

Earlier fungal isolates were also obtained from birds’ nests in mainland New Zealand and the only other known aspergillosis infection in a kākāpō. The environmental samples were obtained using the method of Glare et al. (2013).^18^ Briefly, 20 g of a sample was mixed with 180 mL of 0.01% Triton X-100 and dilutions plated onto Potato Dextrose Agar (Difco), before incubation at 35°C.

All environmental and pathology cultures were re-isolated by streaking out conidial spores and selecting a culture derived from a single haploid conidium for storage and sequencing. These cultures were stored under liquid nitrogen at the ICMP culture collection. Cultures and metadata are available at https://scd.landcareresearch.co.nz/. Matingtype of each *Aspergillus fumigatus* strain was not determined.

### Method details

#### DNA extraction and sequencing

Fungal mycelium was scraped from the surface of an agar culture and ground with liquid nitrogen in a mortar and pestle. For high molecular weight gDNA isolation, the MagAttract HMW DNA Kit (Qiagen, USA) was used following the manufacturer’s protocol. The extracted gDNA was further purified using Agencourt AMPure XP (Beckman & Coulter, USA). The gDNA quality was quantified using a fluorometer, and purity and integrity were assessed with a spectrophotometer and by agarose gel electrophoresis.

#### Genotyping and functional annotation

We deployed an existing SNV and small-indel calling pipeline developed for *A. fumigatus*.^31^ Sequencing reads were mapped to the *A. fumigatus* Af293 reference genome^32^ using bwa mem v0.7.15.^33^ SNVs and small indels were called using GATK HaplotypeCaller v3.6^34^ following that software’s best-practice guide. SNVs at sites with low mapping quality (MQ < 40), evidence of stand-bias (FS > 60) or mapping quality differences between reference and non-reference alleles (MQRankSum < −12) were filtered out before downstream analysis. We obtained functional annotation for all variants using the Ensembl Fungi^35^ variant effect predictor.^36^

### Quantification and statistical analysis

#### Population genetic analysis

Summary statistics from our SNV and small indel data were calculated using vcftools v1.1.16.^37^ Principal component analysis (n = 24 *Aspergillus* strains) was performed on the SNV data using v3.4.4 of the R programming language^38^ and the R packages vcfR v1.11.0^39^ and adegenet v2.1.2.^40^ The genetic distance between isolates was calculated using the bitwise.dist function from poppr v2.8.6.^41^

#### Genome assembly

Isolate ICMP 23423, the isolate from which we obtained the largest number of high-quality sequencing reads, was chosen as the representative isolate of the outbreak strain. A genome assembly was produced using SPAdes v3.11.1,^42^ combining assemblies with kmer values {33, 55, 77, 99, 127}. The quality of the resulting assembly was determined using QUAST v4.0^43^ and compared with the Af293 reference genome.^32^ Copies of putative virulence genes were located using exonerate v2.2.0.^44^

#### Phylogenetic analyses

Whole-genome SNP data were converted into relaxed interleaved Phylip format. Maximum-likelihood phylogenies (n = 247 *Aspergillus fumigatus* genomes) were constructed to assess sequence similarity between isolates using rapid bootstrap analysis over 5000 replicates using the BINGAMMA model of rate heterogeneity in RAxML v8.2.9. Phylogenies were visualized using FigTree v1.4.2.

## Data Availability

• All sequencing reads of *Aspergillus* strains have been deposited at NCBI under NCBI Bioproject: PRJNA726267. Accession numbers are listed in the key resources table. • All original code is available at https://github.com/dwinter/kakapo_aspergillosis and has been deposited at Zenodo and is publicly available as of the date of publication. DOI is listed in the key resources table. • Any additional information required to reanalyse the data reported in this paper is available from the lead contact upon request.

## Supplemental information

Table S1. Aspergillosis outcomes and the infecting strains of *Aspergillus* (where known) for Kākāpō in 2019, relating to Figures 1 and 2

Table S2. Aspergillus strain metadata table, relating to Figures 1 and 2
